# Metagenomics: An Effective Approach for Exploring Microbial Diversity and Functions

**DOI:** 10.3390/foods12112140

**Published:** 2023-05-25

**Authors:** Nguyen Nhat Nam, Hoang Dang Khoa Do, Kieu The Loan Trinh, Nae Yoon Lee

**Affiliations:** 1 Biotechnology Center, School of Agriculture and Aquaculture, Tra Vinh University, Tra Vinh City 87000, Vietnam; 2 NTT Hi-Tech Institute, Nguyen Tat Thanh University, Ward 13, District 04, Ho Chi Minh City 72820, Vietnam; 3 Department of BioNano Technology, Gachon University 1342 Seongnam-daero, Sujeong-gu, Seongnam-si 13120, Republic of Korea; nylee@gachon.ac.kr

**Keywords:** metagenomics, next-generation sequencing, microbial diversity, food safety

## Abstract

Various fields have been identified in the “omics” era, such as genomics, proteomics, transcriptomics, metabolomics, phenomics, and metagenomics. Among these, metagenomics has enabled a significant increase in discoveries related to the microbial world. Newly discovered microbiomes in different ecologies provide meaningful information on the diversity and functions of microorganisms on the Earth. Therefore, the results of metagenomic studies have enabled new microbe-based applications in human health, agriculture, and the food industry, among others. This review summarizes the fundamental procedures on recent advances in bioinformatic tools. It also explores up-to-date applications of metagenomics in human health, food study, plant research, environmental sciences, and other fields. Finally, metagenomics is a powerful tool for studying the microbial world, and it still has numerous applications that are currently hidden and awaiting discovery. Therefore, this review also discusses the future perspectives of metagenomics.

## 1. Introduction

The microbial world was first discovered by Leeuwenhoek using his signature invention, the microscope [1]. The discovery of microbes triggered extensive research aiming to develop methods that could be used to culture different microorganisms. The first culture method was developed by Robert Koch and was called the solid culture media method [2]. Since then, various bacteria have been identified and explored in terms of classification, biological applications, and evolution. The taxonomic status of the microbiome has changed dramatically due to the utilization of 16S ribosomal RNA (rRNA) sequences, which has resulted in the recognition of the archaea group [3]. The 16S rRNA-based phylogenetic marker has been proposed as a crucial tool in taxonomic studies of microorganisms [4], and 16S rRNA continues to be an effective tool in microbial research today [5].

However, not all molecular studies investigating microorganisms have relied solely on 16S rRNA. For example, Handelsman et al. used genomic fragments in environmental samples to clone *E.coli* and explore new mechanisms as well as antibiotic features [6]. Consequently, they proposed the term “metagenome”, which refers to a collection of genomes in the samples for studying cloning and functional analyses. The advent of sequencing techniques has accelerated the development of microbial studies based on the 16S rRNA and whole genomes. The term “metagenomics” has been used to describe studies examining the genomic data of microorganisms, and it can be divided into amplicon and shotgun metagenomics. Amplicon metagenomics studies typically explore microbial diversity, while shotgun metagenomics research is mainly focused on mining functional genes and metabolisms [7,8]. Specifically, targeted gene-based metagenomics is used to obtain portions of each of the microbes in environmental samples, such as soil, water, air, and ice samples [9,10]. The results of shotgun metagenomics also include taxonomic diversity, as in amplicon metagenomics, and they reveal different metabolisms in the microbial community that can be inferred from whole genome sequences [8]. The microbial diversity that has been found in different environments has motivated further studies into the applications of microbial presence in agriculture, food, and pharmacy industries, in addition to human health [11,12,13,14,15]. Shotgun-metagenomics-associated genomic studies have shown a high potential for identifying new bacteria and viruses as well as predicting the major metabolisms in the surveyed environments [16,17,18]. The emergence of metagenomics has provided new paths for exploring the microbial world, which still contains hidden areas.

**Figure 1:**
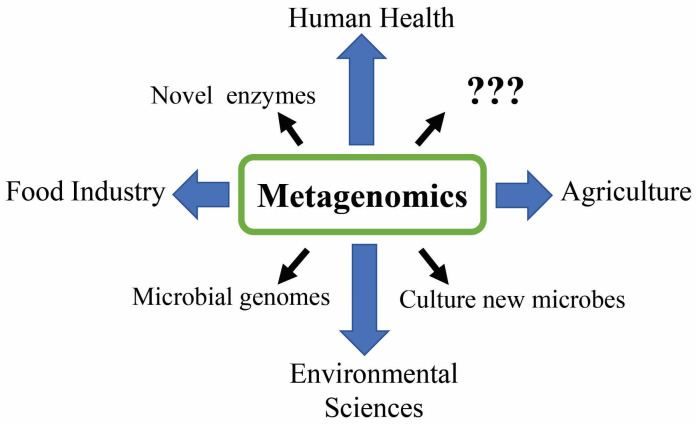
Contributions of metagenomics to various fields. The question marks represent the undiscovered aspects of metagenomics.

Previous reviews have summarized various aspects of metagenomics, including reviews of the methods and pipelines in metagenomics [19,20,21], as well as reviews of different applications of metagenomics in various fields [22,23,24]. In the present review, we summarized the principles of metagenomics using the latest approaches. We also reviewed recent metagenomic studies on human health, agriculture, food industry, environmental sciences, etc., along with their applications. Although previous metagenomic studies have reported significant results, there are still potential applications of metagenomics awaiting discovery. Therefore, we also discuss future perspectives on metagenomics (Figure 1).

## 2. Fundamentals of Metagenomics

**Figure 2:**
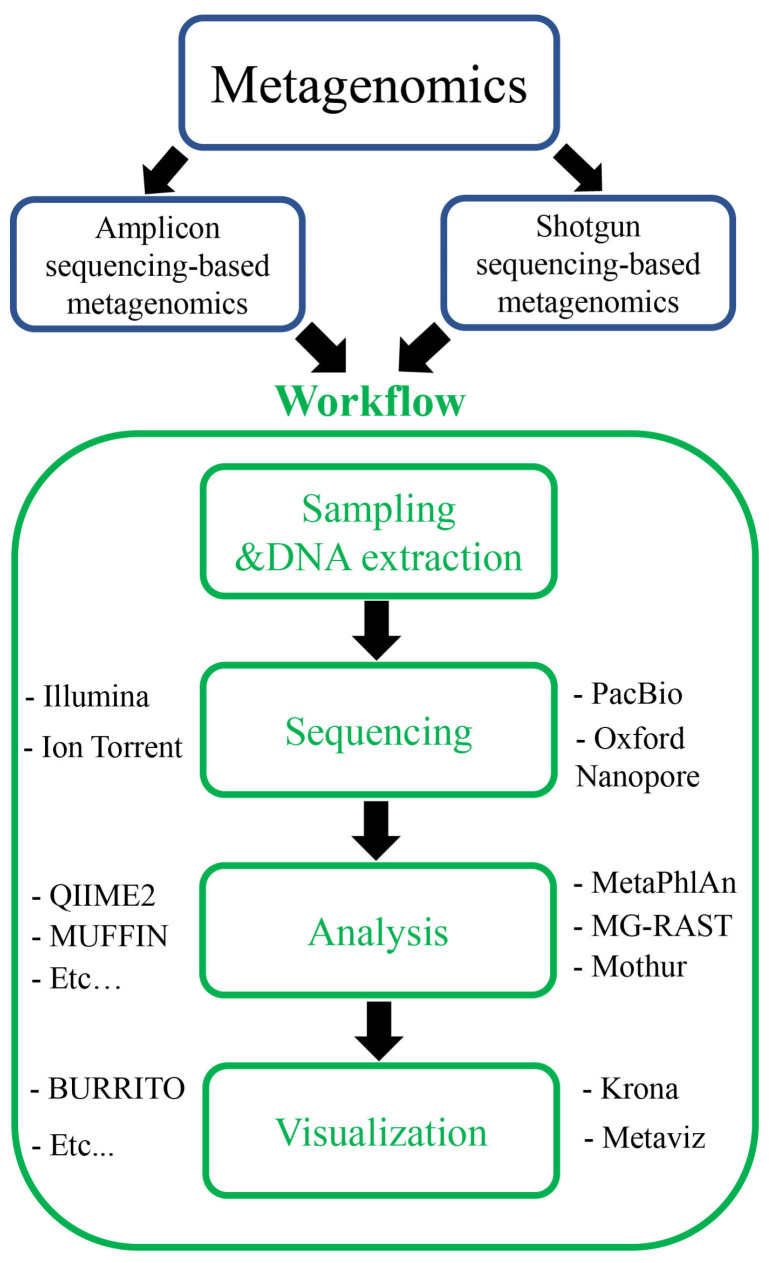
Classification (blue rectangles) and workflow (green rectangles) of metagenomics using different sequencing platforms and bioinformatics tools.

Metagenomics is the study of genomic and genetic data inferred from environmental and clinical samples. Metagenomics can be divided into two groups based on the types of data used: amplicon or targeted gene data and shotgun or untargeted gene data inferred from amplicon and shotgun sequencing, respectively (Figure 2). The data of amplicon metagenomics are amplified sequences of marker genes that include 16S/18S/26S rRNA and intergenic transcribed spacer (ITS) [20,25]. Meanwhile, the data of shotgun metagenomics include all DNA sequences in the samples. Previously, Zhang et al. attempted to classify metagenomics into functional and sequencing metagenomics [26]. In that classification, the field of studies on the discovery of new functional genes and related bioactive substances was called functional metagenomics. Meanwhile, sequencing metagenomics was used to explore the diversity of the microbial community. On the other hand, Breitwieser et al. used the term “metataxonomics” for amplicon sequencing data and “metagenomics” for shotgun sequencing data [27]. Although different terminologies have been proposed for metagenomic classification, metagenomic studies are based on two types of data: amplicon and shotgun data. Therefore, in this study, we used the terms “amplicon metagenomics” and “shotgun metagenomics” for amplicon sequencing and shotgun sequencing data, respectively.

Although the data are different, the process of metagenomics consists of four main steps: sampling and DNA extraction, sequencing, analysis, and visualization (Figure 2). Wajid et al. compared these steps to the process of composing music, in an analogy intended to make the concept of metagenomic research more understandable to an unfamiliar readership [28]. Metagenomic studies often contain a continuous sequence of steps, in which the previous steps decide the outcomes of downstream steps. The samples for metagenomics research are collected directly from the field, so care must be taken care to prevent contamination from other sources. DNA extraction should also be conducted carefully to limit any impurities from the host DNA. In previous studies, specific protocols were developed for different samples such as human fecal samples, tropical soils, and plant tissues [29,30,31]. Meanwhile, commercial kits for metagenomics have also been developed such as FastDNA™Spin Kit for Soil (MP Biomedicals, Irvine, CA, USA), FavorPrep™ Soil DNA Isolation Mini Kit (Favorgen Biotech, Taiwan, China), MagAttract PowerSoil DNA KF Kit (Qiagen, Redwood, CA, USA), PureLink MicrobiomeTM Purification Kit (ThermoFisher Scientific, Waltham, MA, USA), and ZymoBIOMICS 96 Magbead DNA Kit (ZymoBIOMICS, Irvine, CA, USA). The availability of different kits and protocols triggered comparison studies examining the effectiveness of those methods for DNA extraction [32,33,34,35]. The outcomes of those studies suggested that the DNA extraction efficiency and selection of extraction protocols depended on the sample types used. Therefore, suitable protocols or kits should be considered and applied to obtain good results from metagenomic studies.

The data for metagenomic studies generally originate from DNA sequences in different environments, and these are generated using next-generation sequencing methods such as Illumina, PacBio, and Oxford Nanopore Technologies (ONT) [36]. In particular, the Illumina platform resulted in a read length of up to 300 bp, while PacBio and ONT platforms can yield long reads over 1000 bp in length. In contrast to shotgun metagenomics, in which the DNA of sufficient yield can be used for sequencing immediately, the DNA samples for amplicon metagenomics should be amplified with specific primers for targeted genes such as 16S/18S/26S rRNA and ITS [7]. In metagenomic studies of bacteria and archaea, 16S rRNA is commonly used. For fungal and eukaryotic diversity, the small subunit (SSU) rRNA (18S), large subunit (LSU) rRNA (26S), and intergenic transcribed spacer (ITS) have been used [37,38,39]. In amplicon metagenomics, the lengths of the target genes (i.e., 16S, 18S, 26S rRNA, and ITS) are always greater than the sequencing outcomes of the Illumina platform; therefore, partial sequences of target genes have been amplified and used for further analyses. Previous studies have revealed conserved areas and the effectiveness of variable regions in the 16S rRNA [40,41]. For example, primer pairs have been designed based on the conserved regions and can be applied as universal primers for 16S rRNA [40]. Nine hypervariable regions, named V1 to V9, have been found to have specific properties [41]. Various regions (i.e., V1–V2, V3–V4, and V3–V5 regions) have been used to explore bacterial communities at familial, genus, and species levels [42]. Therefore, the selection of regions in the target gene should be based on the particular aims of a given study. The limitations of amplicon metagenomics can be solved using shotgun metagenomics, in which all DNA fragments are sequenced, and the entire length of the target gene can be recovered. However, the assembly process for short reads to complete the whole genes still remains a challenge because the target genes are universal and contain highly conserved regions. The long-read sequencing technique (i.e., PacBio and ONT) can overcome the limitation of short-read sequencing. However, the long-read sequencing platform approach is limited by its high cost and unstable sequencing quality. Oxford Nanopore Technologies has developed a new version for long-read sequencing techniques that produces higher-quality sequencing data (https://nanoporetech.com/q20plus-chemistry, accessed on 8 March 2023). This could reduce the cost and boost long-read-based metagenomic studies in the future.

After the sequencing step, the metagenomic data are processed through the following steps: (1) quality control of reads, (2) assembly/binning, (3) taxonomic/functional profiling, and (4) data visualization. In the first step of quality control, low-quality reads are removed from the data. Then, the remaining reads are assembled to make contigs or mapped to reference genomes. The assembly/binning results are used to predict taxonomic classification and functional mechanisms through comparison to different databases. Finally, all outcomes are visualized to provide details into the microbial composition or potential functions on the microbes. In addition to the emergence of metagenomic studies, different bioinformatic tools have been developed to effectively assist in the analysis process [21]. Wajid et al. have summarized the tools for each analysis step, including 86 tools for quality control, 48 software for assembly, 13 tools for binning, 69 tools for taxonomic classification, 27 tools for gene and functional prediction, 12 tools for metabolic profiling, 15 tools for data visualization, and 4 and 15 databases for microbial taxonomy and functional profile, respectively [28]. Different databases have previously been built for metagenomics including MetaGeneBank for human fecal specimens, Ani-malMetagenome DB, Marine Metagenomics Portal (https://mmp2.sfb.uit.no/, accessed on 8 March 2023), MGnify, TerrestrialMetagenomeDB, and MPD (a pathogen genome and metagenome database) [43,44,45,46,47]. In amplicon metagenomics, the main output is microbial composition, so the tools were built to clean, cluster, and quantify data, such as VSEARCH, DADA2, and Deblur [48,49,50]. Specifically, DADA2 can identify exact amplicon sequence variants and produce fewer false positive sequence variants than other methods [49]. Similarly, Deblur can denoise sequences and be applied to large datasets [48]. However, only single-end reads can be used as input data for Deblur. Moreover, taxonomic classification is an important output of amplicon metagenomics, which can be conducted quickly using Kraken 2 [51]. For shotgun metagenomics that are mainly focused on functional analysis, databases play an important role, and KEGG (Kyoto Encyclopedia of Genes and Genomes) and COG (Clusters of Orthologous Groups of proteins) databases have been built [52,53]. Additionally, the tools for functional profiling and prediction were developed, including PICRUSt2, MEGAN, GeneMark-HM, and Prokka [54,55,56,57]. Another database, namely Functional Annotation of Prokaryotic Taxa (FAPROTAX), contains software that can be used to convert taxonomic profiles to putative functional profiles [58]. A previous study showed that FAPROTAX is a promising tool for predicting the function of bacteria in soil samples [59]. Similarly, FAPROTAX analysis of soil microbiota revealed a correlation between functional groups and physicochemical properties in mangrove soil [60]. Although various tools have been developed for metagenomic analysis, there are still challenges that arise during the analysis process, which were critically discussed by Breitwieser et al. [27]. Aside from the development of new bioinformatic tools, some previous tools have been upgraded with more effective results (Table 1).

For example, MetaPhlAn 4 employed a database of more than one million prokaryotic reference genomes to achieve comprehensive metagenomic taxonomic profiling [61]. HUMAnN 3, StrainPhlAn 3, PanPhlAn 3, and PhyloPhlAn 3 exhibit effectiveness in the strain-level, phylogenetic, taxonomic, and functional profiling of microbial communities [62]. Further, QIIME2 is a good bioinformatics platform for analyzing and visualizing metagenomic data [63,66,67]. Recently, the combination of DIAMOND + MEGAN and the release of MeganServer have been shown to provide a user-friendly and effective platform for exploring the taxonomic and functional analysis of short and long metagenomic sequence data [64,65]. For food-based metagenomics, Kobus et al. introduced a novel computational method, called MetaCache, which could be divided into AFS-MetaCache (based on C++) and MetaCacheSpark (based on Apache Spark), and which exhibited fast running, low false-positive rates, and high quantification accuracy features [68]. Moreover, ARG-ANNOT (Antibiotic Resistance Gene-ANNOTation) and DeepARG, which was the result of a machine-learning methodology, could detect the antibiotic resistance features of microbes in the raw reads of metagenomic data from fermented food [69]. Tools have also been created for illustrating the results of metagenomic studies, such as Metaviz, Krona, and BURRITO, and these provide interactive illustrations of metagenomic results [70,71,72].

The availability of various tools has led to the development of pipelines for metagenomic analyses [73]. Garfias-Gallegos et al. created an effective pipeline as the first step toward quality control in the final visualization of the metagenomic data [74]. Similarly, the Omnibus metagenome-wide association study with robustness (OMARU) pipeline was developed to explore the relationship between microbiomes and disease pathophysiology [75]. Previously, Navgire et al. summarized various pipelines for metagenomic studies of crops [19]; these pipelines were applied to different plants, such as rice, sugarcane, peanut, and wheat, and they could serve as a model for further studies in different areas. Another advancement in metagenomics is the availability of web-based tools that allow users to upload data and wait for outcomes; this reduces the time and cost for scientists who do not have enough facilities (i.e., supercomputers) for analysis in their lab. Some popular websites for metagenomics are MG-RAST, EBI MetaGenomics, IMG/M, and EDGE platform [76,77,78,79]. In these web-based platforms, the analysis pipeline commonly consists of quality control of reads, assembly, function prediction, taxonomic prediction, and the visualization of results steps using various tools and databases. Users can simply upload the metagenomic data to the platforms and select appropriate options for research purposes. The continued advent of improved technology and the availability of bioinformatic tools have allowed for metagenomics studies to be conducted effectively and easily; however, suitable strategies inferred from the aims of the studies should be considered to obtain the best outcomes.

## 3. Metagenomics in the Food Industry

Among the current types of foods that are eaten often, fermented foods such as kimchi, yogurt, kefir, kombucha, tempeh, and sauerkraut rely on the presence of different microbes to achieve specific flavors [80,81]. Previously, microbial composition in foods has been checked using culture methods, which are limited in their ability to find unculturable bacteria [82]. The availability of a metagenomic approach has provided a powerful tool for exploring microbial communities in food [13,83]. For example, the bacteria in biofilms, including *Pseudomonas* spp., *Acinetobacter* spp., *Leuconostoc* spp., *Lactobacillus* spp., and *Streptococcus* spp., in food processing plants have been characterized based on 16S rRNA sequencing data [84]. In addition to amplicon metagenomics, functional metagenomic studies examining food have revealed potential applications in the food industry, such as the identification of novel enzymes for processing foods [85]. In the field of fermented foods, metagenomic analysis has resulted in new knowledge that is used for exploring, ensuring, and improving food quality [86,87]. For instance, different bacterial compositions in fermented bamboo shoots (Tuaither), soybeans (Bekang), and pork fat (Sa-um) have been surveyed using a metagenomic approach [88]. The most abundant types of bacteria in Tuaither, Bekang, and Sa-um were *Lactobacillus*, *Staphylococcus*, and *Clostridium*, respectively. These findings have revealed the correlation between the types of ingredients used and the abundance of bacteria in fermented food. Aside from indicating the diversity of microbial communities, metagenomics has enabled the differentiation of the metabolic pathways of biogenic amines caused by various microbes during the fermentation of *Brassica juncea* [89]. The connection between the predominance of microbes and metabolism was also investigated in Yucha, which is a fermented food composed of fresh fish and cooked rice that is popular in China [90]. For traditional foods such as kimchi, yogurt, kefir, kombucha, tempeh, and sauerkraut, which were developed thousands of years ago based on the experiences of our ancestors, metagenomic studies shed light on the factors (i.e., microbes and enzymes) and process involved in achieving the distinct flavors of these foods. For instance, one study found that three genera of *Leuconostoc*, *Lactobacillus*, and *Weissella* were dominant in kimchi [91]. In addition to bacteria, the existence of bacteriophages was recorded, suggesting an influence on the microbial community during fermentation. Another study on kimchi at the industrial scale revealed the presence of *Leuconostoc mesenteroides*, *Lactobacillus sakei*, *Lactobacillus plantarum*, and *Weissella koreensis* [92]. Another larger study based on 88 kimchi samples prepared at different locations, in different seasons, with various ingredients, and by several preparation methods revealed that the bacterial communities in kimchi were easily affected by many factors, while location did not have a significant effect [93]. These results have contributed to explaining the different tastes of fermented foods originating from households and industries. The outcomes of metagenomics have allowed for further research into applications related to the bacterial communities in kimchi [94,95]. Park compared the conventional conditions and CO_2_-rich environments during kimchi fermentation [94]; the results showed the effectiveness of CO_2_ addition on the quality, metabolisms, and alteration of microbes in kimchi products. Another study on treating kimchi with light-emitting diodes (LEDs) indicated that different wavelengths of the LED source could alter the microbial composition [95]. Consequently, the metabolomic pathways also change. Such results have exhibited the potential of using LEDs to control the quality and create new tastes in fermented foods in the future. Similarly, metagenomics has been used to sheds lights on the microbial world of other fermented foods, including yogurt, kefir, kombucha, tempeh, and sauerkraut [96,97,98,99,100,101,102,103,104]. In addition to exploring the role of microbes in different fermented foods, the metagenomic approach allows to identify the functional roles of bacteria during food production. Consequently, producers can alter the process to optimize product quality and reduce food waste [87].

The ability of metagenomics to provide the fundamentals of microbial communities has enabled the application of metagenomic studies to food safety in which the presence of foodborne microbial pathogens and phytopathogenic fungi was detected through high-throughput sequencing methods [105]. Furthermore, metagenomics revealed an effectiveness in detecting plant pathogens which should be monitored for global food security [106]. Previously, Tatsika et al. examined the bacterial composition of ready-to-eat vegetables using 16S rRNA and revealed different bacterial diversities in various types of salads [107]. Fortunately, there was no record of foodborne pathogenic taxa in the surveyed samples. Furthermore, their study showed that the removal of microbes on vegetables can be successfully conducted using washing methods [107]. The microbiomes on the surface of fruits such as white guava, passion fruit, and papaya harvested in Northern Argentina were also explored using shotgun metagenomics [108]. The results revealed the presence of bacteria, yeasts, and filamentous fungi on the surface as well as unidentified species, suggesting further studies examining intrinsic species in plants. The microbial composition in apple fruits before and after processing was investigated based on 16S rRNA sequences [109]. The results exhibited a reduction of microbes after processing; however, some bacteria such as *Pseudomonas* and *Ralstonia* still survive on apples, suggesting a need for monitoring these taxa on fruits. One study sequences the total RNA content in the food ingredients to test the correlation between shifts in microbiomes and contaminants [110]. Moreover, RNA sequencing allowed for detection of the viability of microbes in food. The long-read metagenomics sequencing method showed high effectiveness for the detection of Shiga toxin-producing *Escherichia coli* (STEC) contamination in water at 10^3^ CFU/mL (68 reads), suggesting its potential applicability to other foodborne pathogens [111]. In addition to identifying microbes in foods, the metagenomic approach has allowed for the screening of microbial existence in factories, tools, and any state of production [112]. Although methods and bioinformatics tools have been developed for exploring microbiomes in fruits, a suitable strategy should be selected for the aims of study. Previously, Jo et al. compared DNA and mRNA libraries and three analytical methods for microbial diversity in overwintering pepper fruits and demonstrated that library types, analytical methods, and proper databases contribute to the achievement of microbiome study [113].

## 4. Metagenomics in Human Health

The metagenomic approach has revolutionized studies investigating the human microbiome originating from different parts of the human body, such as the skin, oral cavity, lung, and intestine [114,115,116,117]. A recent review described the advancements that have been made in technologies for exploring microbial DNA in human samples wherein host DNA depletion and microbial DNA enrichment were summarized [118]. Previously, an efficient strategy called MetaGeniE was developed to identify microbes with high specificity and sensitivity [119]. In particular, the MetaGeniE pipeline includes two parts: Read-Reduct and Patho-Detect. The former part results in high-quality reads after conducting filtration using PRINSEQ [120], BWA [121], and STAMPY [122]. In the Patho-Detect part, the filtered reads are aligned to genomic databases to identify microbial composition using BLAT [123]. Moreover, the mBodyMap containing 14,401 metagenomes related to 22 body sites and 56 human diseases was introduced as a useful database for further research into human microbiomes and related diseases [124]. Consequently, various studies have reported applications of metagenomics for surveying the pathogens in the human body, which is a necessary aspect of formulating strategies for public health [125,126,127]. For example, Malla et al. summarized the sequencing technologies and bioinformatic tools that were used to explore the connection between microbiomes, human health, and diseases [125]. Further, applications of metagenomics as new therapeutic approaches for diagnosis and treatment are covered and discussed. Similarly, Ko et al. outlined the use of metagenomics for pathogen surveillance (i.e., the detection of pathogens and antimicrobial resistance genes of microbes in global sewage) [126]. Further, untargeted metagenomics has revealed the dynamics of microbial communities in cystic fibrosis patients and antibiotic-resistance genes [127]. Another study based on nanopore metagenomics showed that it could be used as a rapid and effective diagnostic tool for pneumonia pathogens [128]. Similarly, various viruses such as rhinovirus, coronavirus, parainfluenza, parechovirus, metapneumovirus, and influenza virus have been identified in clinical specimens using BLASTN [129], bowtie2 (version 2.2.5) [130], and PCR validation [131]. ONT sequencing-based metagenomics exhibited the rapid and accurate characterization of results of the bacterial communities in lower respiratory infections with high specificity and sensitivity [132]. Similarly, nanopore-sequencing-based metagenomics showed 83% sensitivity and 100% specificity for detecting the influenza virus in respiratory samples [133]. A metagenomic study on tuberculosis patients demonstrated the relationship between the pulmonary microbiome and its clinical characteristics [134]. Another review demonstrated the interactions between bacterial communities and human immunology through the developmental stages of infancy, childhood, and adulthood [135]. The results of these prior studies prove the advantages and effectiveness of next-generation sequencing-based metagenomics as a diagnostic tool for lower-respiratory-tract infections and their potential therapy for respiratory diseases [136].

In the human microbiome, the oral microbial communities have also been screened [137]. From the current oral microbiome data, about 56,213 metagenome-assembled genomes have been obtained, in which 64% of the sequences were not previously reported [115]. A survey of 47 children with dental caries and healthy dentition revealed that *Prevotella* spp., *Streptococcus mutans*, and the Epstein–Barr virus were all correlated with caries [138]. An interesting metagenomic study on the oral microbial communities in ancient humans revealed that *Anaerolineaceae* bacterium was dominant [139]. The results of that same study also indicated a significant shift in resistance to antibiotics from prehistoric humans to modern ones. Aside from providing the taxonomy and composition of the microbiome, metagenomics supplies new evidence of associations between human genetics and oral microbiomes [140]. Specifically, five loci in the human genome, including *APPL2*, *SLC2A9*, *OR11H1*, *LOC105371703*, and *MGST1*, were related to oral microbes. Moreover, the host genetics were found to be responsible for dental diseases instead of the oral microbiome, suggesting that specific therapies should be designed for each individual case. However, a recent review has emphasized the impact of oral microbiota on oral diseases [141]. The effects of oral bacteria are based on the release of pro-inflammatory cytokines. The availability of data facilitated metapangenomics of the oral microbiomes, which showed a correlation between the genomic diversity of the oral bacteria and environmental features [142]. An additional application of metagenomics for oral microbiomes is the mining of new genes. Previously, unknown acid-tolerant genes were identified in dental caries patients, suggesting the feasibility and efficiency of metagenomics for finding functional genes [143].

Most metagenomic studies on human microbiomes have focused on gut microbiota, which exhibits correlations to digestion, immunity, and diseases [144,145,146,147,148,149,150]. For example, a large-scale analysis of the gut microbiomes in Parkinson’s disease (PD) patients revealed microbes responsible for dysbiosis and genes related to the PD mechanism [151]. These findings have provided the foundations for further studies investigating the treatment of PD using different microbial compositions. Recently, bioinformatic strategies and technological developments with various benefits and limitations for gut microbiomes have been summarized and discussed [152]. The popularity of metagenomic studies and bioinformatic tools (i.e., CheckM v1.0.11 [153], dRep v2.2.4 [154], Mash v2.1 [155], FastANI v1.1 [156], VirSorter v.1.0.5 [157], VirFinder v.1.1 [158], CONCOCT v1.1.0 [159], GTDB-Tk v1.0.2 [160], and Prodigal v2.6.3 [161]) has led to the formation of human gut microbiome database, such as the Human Reference Gut Microbiome (HRGM), gut MEtaGenome Atlas (gutMEGA), Unified Human Gastrointestinal Protein (UHGP), and Metagenomic Gut Virus catalog [162,163,164,165]. The sequence data in these databases can be easily downloaded, and they serve as useful taxonomic references for conducting metagenomics research on humans under various conditions. For example, recent findings on microbiomes have revealed significant differences between monks and control subjects [166]. Specifically, monks who practiced long-term meditation had abundant *Prevotella*, *Bacteroides*, *Megamonas*, and *Faecalibacterium* species. In addition, several pathways, such as those of glycan biosynthesis and lipopolysaccharide biosynthesis, were predicted to be significantly higher in person who practice meditation. By contrast, the monks had lower levels of cholesterol and apolipoprotein B, which are factors that potentially affect human health. These results indicate the benefits of meditation, which helps to reduce stress and enhance the immune system. Another review examining the health benefits of adding probiotic microorganisms showed positive impacts on human health and several diseases [167]. One of the possible outcomes of a metagenomic study is to find correlations between microbiomes and cancers. Ng et al. summarized the factors related to colorectal cancer carcinogenesis and discussed potential methods for the diagnosis and treatment of colorectal cancer [168]. Another application of metagenomics is to identify drug efficacy and toxicity through bacterial compositions. A study on 4198 individuals revealed the small and large effects of different drugs to gut microbiome [169]. A recent study on the human microbiomes of patients with gastrointestinal symptoms caused by the severe acute respiratory syndrome coronavirus 2 (SARS-CoV-2) showed a decrease in bacterial strains in these COVID-19 patients [170]. Moreover, after recovery, the compositions of the gut microbiomes in COVID-19 patients have been found to be quite different from those of the control subjects. A similar study on COVID-19 patients provided detailed information on the connection between the microbiome and disease symptoms [171]. Specifically, the opportunistic pathogen bacteria increased significantly while the protein metabolism and carbohydrate-oriented pathways were overexpressed in COVID-19 patients. Two independent research teams reported coevolution and codiversification between the gut microbiomes and human hosts [172,173]. An investigation of the gut microbiota of children suggested a new target for better growth in low- and middle-income countries: the genetic functions of microbes [174].

Another application of metagenomics is in the exploration of the presence of viromes and their therapeutic utilization for human health [175]. Consequently, the presence of the viromes and interaction with other viruses in the human body resulted in the development of therapeutic applications such as fecal microbiota transplantation, phage-based therapy, and oncolytic therapy [175]. A previous review summarized the detection of various viruses and bacteriophages from the human microbiome [176]. The presence of viruses was found to be related to inflammatory bowel disease, diarrhea, obesity, and diabetes [177,178]. These findings indicate the potential for developing diagnostic and therapeutic measures for viruses-related diseases. For example, bacteriophages have been shown to be effective in the treatment of patients infected with *Clostridium difficile* [179,180]. Although the applications of human virome were suggested and tested, some cautions should be addressed before wide applications to human beings, such as clinical protocols, validation of the outcomes, and details of the mechanistic interactions [175,176].

## 5. Metagenomics in the Environmental Sciences

Soil is an important part of the Earth that contains organic and inorganic substrates, as well as provides a living environment for many species. Metagenomics has enabled research into microbial diversity in different soil types [181]. For example, one study on the soil microbial communities in different ecosystems, such as deserts, forests, grasslands, and tundra, revealed a relationship among bacterial composition, functional genes, and the environment [182]. For example, deserts were found to have low taxonomic and functional diversity compared to other surveyed locations. Further, genes related to osmoregulation and dormancy were abundant in the desert samples. By contrast, a low number of antibiotic-resistant genes was noted from the desert bacterial community, suggesting minor competition in the desert biome. Similarly, the results of Arctic soil microbiomes from different depths have indicated a significant decrease in microbial biodiversity and notable change in the functional genes with depth [183]. Another finding is that the microbiomes in tropical rainforest soils were less diverse than those in grasslands and agricultural soils [184]. Metagenomic research has also provided an overview of the alterations in microbiomes in soils over time. Observation of the microbial compositions before and after building an oil pipeline indicated a dramatic increase in Microcoleaceae, Pinaceae, and Williamsiaceae, along with a notable decrease in Psychromonadaceae and Leuconostocaceae in samples from 2016 compared to those from 2013 [185]. Similarly, an increase in *Nitrosospira* and *Sulfuricella* genera, which is related to cadmium tolerance, was detected in soil samples with cadmium contamination in comparison to non-contaminated soil [186]. Changes in the microbial communities in total petroleum hydrocarbon (TPH)-contaminated soil were explored, in which the portion of TPH-degrading bacteria (i.e., beta-, gamma-, and delta-proteobacteria) increased by 9% compared to the control sample [187]. Another study on mercury-contaminated soils revealed differences in microbes in the rhizospheric and bulk soils [188]. The rhizospheric soils had a high abundance of Proteobacteria, whereas Actinobacteria and Alphaproteobacteria were the majority in the bulk soil. This result revealed selective effects in contaminated soils which are useful for bioremediation. A recent observation of the microbial composition of the rhizospheres of coastal plants revealed a correlation between the soil minerals and bacterial communities, suggesting that microbes may potentially play roles in stress resistance in coastal plants [189]. The availability of soil metagenomic data resulted in the formation of the largest publicly available sequencing dataset that was managed and updated annually by the National Ecological Observatory Network (NEON) [190]. The results of soil metagenomics have indicated not only the diversity of microbes but also potential ways to clean polluted soils.

In addition to the soil environment, metagenomics has been used to assess the microbial communities in water [191]. Various water bodies, such as oceans, lakes, mangrove ecosystems, rivers, and canals, have been used for metagenomic research [192,193,194,195,196,197,198,199,200]. Further, results obtained using metagenomics have uncovered potentially novel deep-ocean microorganisms and diverse metabolic strategies [201]. The available marine biome data were combined to establish the MarineMetagenomeDB, which provides an effective resource for further metagenomic studies [202]. In another work, the presence of microorganisms in wastewater was studied to formulate a suitable recycling method [203]. A recent study on poly-contaminated groundwater showed that the presence of Burkholderiales can degrade various contaminants, thus indicating a suitable method for bioremediation [204]. Metagenomic tools have allowed for the assessment of the microbial communities in drinking water, which has become a useful solution for quality monitoring [205,206]. In addition to common bacteria in polluted water, the outcome of their study revealed that *Arcobacter* and *Aeromonas* could be used as pollution indicators for fecal pollution source tracking [205]. In addition to bacterial detection, metagenomics could also be used to explore the presence of viruses in drinking water [207].

Similar to the soil and water environments, the air also contains microbes. However, metagenomic studies of air samples face some challenges regarding microbial density, standardized methodologies, and bioinformatics tools [208]. Fortunately, the advent of new technologies for sample collections (i.e., the TOP filter system), DNA sequencing (i.e., Illumina, PacBio, and Oxford Nanopore Technologies platforms), and analysis pipelines (i.e., MEGAN and QIIME2) have allowed for the application of air metagenomics to explore metabolic pathways, bioremediation methods, and biogeochemical cycles [20,209]. Although the low biomass feature of the air environment has resulted in a lower number of microbes than in soil and water media, metagenomic research has revealed differing abundances of microbial communities [210]. Metagenomic studies examining the air environment at different locations revealed airborne microbiome alterations. A metagenomic analysis of 3226 air samples revealed both positive and negative correlations between anthropogenic activities and airborne communities [211]. Moreover, *Acinetobacter*, *Corynebacterium*, *Mycobacterium*, and *Staphylococcus* genera were found to be positively related to the mortality rates of patients with respiratory diseases. Further, airborne pathogens were more likely to emerge from the surfaces of the human body. Another study examining 370 air samples around the world indicated that the features of the surface environment determined the abundance of airborne bacteria [212]. Additionally, the aerial environments and the microbes in the nearby ecosystems could impact the variation of global airborne bacteria. A notable study on 789 metagenomes collected from a single site during a one-year period has advanced our understanding of the correlations between the dynamics of bacterial composition and the Diel cycle [213]. The diversity of the microbes showed daily variations rather than fluctuations of bacterial communities on a daily or monthly basis. Further, among the environmental parameters such as temperature, humidity, and CO_2_ concentration, temperature was found to be the main factor affecting microbial community dynamics. Metagenomic studies on indoor air have also exhibited a diversity of DNA and RNA viruses [214]. Hospital air directly affects patient health and is an important target for metagenomic research. Previous results based on the shotgun metagenomic approach have revealed an abundance of opportunistic pathogens such as *Aspergillus*, *Penicillium*, and *Stenotrophomonas* [215]. Moreover, a multi-drug resistant bacterial strain (i.e., *Stenotrophomonas maltophilia*) has been observed. Another metagenomic study found resistomes in *Staphylococcus*, *Micrococcus*, *Streptococcus*, and *Enterococcus* species in hospital air [216]. Moreover, the higher antimicrobial resistance associated with hospital air compared to urban ambient has been discovered [217]. Collectively, metagenomic research has shown a large diversity of microbes in the soil, water, and air environments. The presence of various microorganisms has triggered the primary application of metagenomics as a tool for the identification of specific microbes. Further, the existence of bacteria can provide necessary initial information for implementing bioremediation in polluted environments.

## 6. Metagenomics in Agriculture

The advantage of metagenomics in defining microbial compositions has made it a useful method for monitoring agriculturally important pathogens and diseases [218]. Functional metagenomics also provides a powerful tool for elucidating the interaction between crops and microbes to increase crop yield and to identify new genes for stress resistance in crops. To benefit from the advantages of metagenomic features, different techniques have been summarized and discussed [15,219]. For example, a review of bioinformatic tools for metagenomic studies of anaerobic digesters has shown that various approaches can be used, such as those involving artificial intelligence and neural network software [220]. Recently, aerial environmental DNA data have been used to monitor pathogens in crop fields [221]. Obviously, sustainable agricultural development requires the results of metagenomic studies [222]. Previously, microbial communities in saline environments have been identified together with three different planting systems, including a conventional system, an aerobic system, and a system of rice intensification (SRI) [223]. As a result, the SRI soil samples exhibited higher species diversity than the other methods. Moreover, varied functional properties were found in all soil samples collected from the three systems, suggesting the effectiveness of different planting systems. A screening of the microbial communities in desert farming systems revealed unexpectedly large diversity [224]. Aside from the diverse composition, unknown bacterial groups were detected, suggesting novel plant microorganism interactions. Another shotgun metagenomic research on the maize rhizosphere showed an abundance of *nif*H, *nif*A, *gro*ES, and *csp*A genes, which can be potentially employed to reduce environmental stress and enhance plant development [225]. Metagenomic studies have also enabled the detection of novel viruses belonging to Betaflexiviridae, Tombusviridae, and Geminiviridae families in maize [226]. Aside from maize, similar metagenomic research has also been conducted for other plants such as wheat, maize, sugarcane, rubber tree, and vegetables [227,228,229,230,231,232].

In addition to crop-based studies, metagenomic research has been applied to farm animals [233]. Glendinning et al. conducted a metagenomic study examining four ruminants, including cow (*Bos taurus*), sheep (*Ovis aries*), reindeer (*Rangifer tarandus*), and red deer (*Cervus elaphus*), and they constructed 391 microbial genomes, out of which 279 records were new taxa [234]. Similarly, Sato et al. assembled 146 genomes from the cattle rumen [235]. The outcomes of such metagenomic research were not only bacterial compositions but also viral information [236]. A recent study on the virus communities in chicken farms has indicated the utility of metagenomics in tracking viral pathogens [237]. Another application of metagenomics is for the determination of the relationship between the microbial community and host nutrition and metabolisms [238]. Further, differences in microbial compositions between domestic and wild animals were addressed, and exhibited a high abundance of antimicrobial resistance genes in the microbiomes of farm animals [239]. A survey on common resistomes showed 201 antibiotic-resistant genes (ARGs) in different animal manures [240]. However, the number of ARGs was reduced significantly in commercial compost. Therefore, it is necessary to upgrade the production progress to minimize the presence of ARGs in agricultural ecosystems. Structures of the microbial communities have also shed lights on ruminal fermentation, which can be employed for further research into plant biomass degradation [241].

For aquatic animals, metagenomics is a powerful tool for exploring the microbial communities necessary to improve water quality, treat wastewater, and prevent diseases. Strategies based on different sequencing methods for exploring microbes in recirculating aquaculture systems have been established, and these have provided fundamentals for further metagenomics related to aquaculture [242]. A previous metagenomic study characterized the microbial diversity in shrimp ponds [243]. Similarly, the core bacterial genera in the gut of shrimps were identified, wherein nine taxa had strong relations to the fast growth of shrimp [244]. This observation has advanced our understanding of how to control the development of shrimp. Metagenomics has also helped assess the efficiency of newly developed technologies for aquaculture ecosystems. For example, a test of the biofloc technology for a shrimp farm revealed that biofloc-based aquaculture had more opportunistic pathogens [245]. Overall, metagenomics has exhibited significant effectiveness in agriculture for pathogen monitoring, antibiotic resistance detection, and quality improvement.

## 7. The Future of Metagenomics: Novel Fields and Future Perspectives

For the fields of biochemistry and biotechnology, metagenomics is a molecular tool that can be used to find new enzymes from microbial communities [246]. The workflows and strategies for mining novel enzymes through functional screening and sequence-based metagenomic approaches have been reviewed in some earlier works [247,248,249]. Specifically, Sung et al. developed an approach for focused identification of the NGS-based definitive enzyme research (FINDER) strategy for the rapid large-scale screening of environmental microbiota and enzymes [249]. A previous review summarized 332 industrially relevant enzymes from unculturable microorganisms [250]. Different enzymes, such as lipases, cellulases, and proteases, have been identified based on metagenomic data [251]. At present, the rapidly increasing volume of metagenomic data is expected to result in more reports on novel enzymes in the future.

For microbial diversity, metagenomic databases are useful sources for identifying new microbes in different environments. Unlike amplicon metagenomics, shotgun metagenomics provides all the DNA sequences in a sample. Therefore, various workflows for reconstructing complete genomes from metagenomic data have been developed [252,253,254,255]. A recent study reported 4142 microbial metagenome-assembled genomes in the horse gut microbiome, of which 4015 records potentially belong to new species [256]. In addition to bacterial genomes, viral genomes were successfully assembled from the metagenomic data [257]. The diatom community was also characterized using the metagenomic approach [258]. Similarly, 24 lichenized-fungal genomes were completed, and these indicated high diversity and dissimilarity in the secondary metabolite biosynthetic gene cluster of lichens [259].

Metagenomics has offered an effective method for observing uncultured microbes at the genetic level in the field of microbiology [260]. Complete genomes of unknown microorganisms have also been completed from metagenomic data. These precise genomic data have enabled the use of different strategies to convert uncultured microbes to cultured ones [261]. The availability of complete genome sequences is necessary to predict the various metabolic pathways that are needed to prepare suitable culture media; then, uncultured microbes could be cultured using different approaches, such as ARG-based isolation, stable-isotope-probing-guided Raman-activated microbial cell sorting, gene-targeted isolation, and reverse-genomics-guided isolation. Although successful isolations of uncultured microbes have been reported, certain challenges remain, including optimization of the culture media, various sizes for the cell sorter, unknown gene expression, and DNA extraction methods.

Lastly, the recent ESM Metagenomic Atlas constructed using artificial intelligence contains more than 617 million protein structures, out of which millions were new compared to the available protein database [262]. This finding has opened new paths to explore metagenomic data using artificial intelligence, which provides powerful assistance for exploring uncovered parts of the scientific world. Obviously, metagenomics has opened new doors for exploring the microbial world on Earth. However, further discovery to achieve a better understanding requires more advancements in terms of sampling protocols, analysis pipelines, databases, and interpretation of the results.

## Figures and Tables

**Table 1:** List of recently upgraded tools and platform for metagenomic analysis.

Tool/Platform	Functions	Year/Reference
MetaPhlAn 4	Comprehensive metagenomic taxonomic profiling	2023/[61]
HUMAnN 3	Efficient and accurate functional profiling	2021/[62]
StrainPhlAn 3/PanPhlAn 3	Nucleotide- and gene-variant-based strain profiling	2021/[62]
PhyloPhlAn 3	Phylogenetic placement and putative taxonomic assignment	2021/[62]
QIIME2	Analysis and visualization of amplicon metagenomic data	2019/[63]
DIAMOND + MEGAN	Taxonomic and functional analysis of short and long metagenomic sequence data	2021/[64]
MEGAN6 and MeganServer	Taxonomic and functional analyses and visualization of metagenomic data	2023/[65]
Kraken 2	Fast taxonomic classification	2019/[51]
